# Antibacterial Activity of combinatorial treatments composed of transition-metal/antibiotics against *Mycobacterium tuberculosis*

**DOI:** 10.1038/s41598-019-42049-5

**Published:** 2019-04-02

**Authors:** L. Z. Montelongo-Peralta, A. León-Buitimea, J. P. Palma-Nicolás, J. Gonzalez-Christen, J. R. Morones-Ramírez

**Affiliations:** 1 Universidad Autónoma de Nuevo León, UANL. Facultad de Ciencias Químicas. Av. Universidad s/n. CD. Universitaria, 66455 San Nicolás de los Garza, NL Mexico; 2 Centro de Investigación en Biotecnología y Nanotecnología, Facultad de Ciencias Químicas, Universidad Autónoma de Nuevo León. Parque de Investigación e Innovación Tecnológica, Km. 10 autopista al Aeropuerto Internacional Mariano Escobedo, 66629 Apodaca, Nuevo León Mexico; 3 Centro Regional de Control de Enfermedades Infecciosas, Facultad de Medicina, Universidad Autónoma de Nuevo León, UANL. Av. Madero and Dr. Aguirre Pequeño s/n, Mitras centro, 64460 Monterrey, Nuevo León Mexico; 4 Laboratorio de Inmunidad Innata. Facultad de Farmacia. Universidad Autónoma del Estado de Morelos. Av. Universidad 1001, Col. Chamilpa, 62209 Cuernavaca, Morelos Mexico

## Abstract

Notwithstanding evidence that tuberculosis (TB) is declining, one of the greatest concerns to public health is the emergence and spread of multi-drug resistant strains of *Mycobacterium tuberculosis* (MDR-TB). MDR-TB are defined as strains which are resistant to at least isoniazid (INH) and rifampicin, the two most potent TB drugs, and their increasing incidence is a serious concern. Recently, notable efforts have been spent on research to pursue novel treatments against MDR-TB, especially on synergistic drug combinations as they have the potential to improve TB treatment. Our research group has previously reported promising synergistic antimicrobial effects between transition-metal compounds and antibiotics in Gram-negative and Gram-positive bacteria. In this work, we evaluated antimycobacterial activity of transition-metals/antibiotics combinatorial treatments against first-line drug resistant strains of *Mycobacterium tuberculosis*. Our data showed that INH/AgNO_3_ combinatorial treatment had an additive effect (bactericidal activity) in an isoniazid-resistant clinical strain of *Mycobacterium tuberculosis*. Moreover, *in vitro* evaluation of cytotoxicity induced by both, the individual tratments of AgNO_3_ and INH and the combinatorial treatment of INH/AgNO_3_ in murine RAW 264.7 macrophages and human A549 lung cells; showed no toxic effects. Together, this data suggests that the INH/AgNO_3_ combinatorial treatment could be used in the development of new strategies to treat resistant strains of *Mycobacterium tuberculosis*.

## Introduction

*Mycobacterium tuberculosis* (*M*. *tuberculosis*) causes tuberculosis (TB), which is the leading cause of death by infectious diseases worldwide, with an estimated 10.4 million new TB cases in 2016^1^. One of the main challenges in TB drug development is that the treatment scheme is a combined regimen, not a single drug. Therefore, the research of new treatments for drug-resistant tuberculosis should be based on novel mechanisms of action relative to the current TB therapy, considering the least amount of undesirable interactions between drugs and the least possible number of side effects; this will lead to obtaining a treatment with a high therapeutic potential in patients with MDR-TB^2^. Consequently, in recent years the development of metallo-pharmaceuticals has increased since they are compounds that include metals due to their therapeutic action, and currently belong to a class of promising antimicrobial compounds aimed to overcome resistant strains^3–10^. Transition metal species, and especially silver compounds, lie among the most studied metallo-pharmaceuticals^11,12^.

Recent literature has reported on the antimycobacterial activity of transition-metals (ions, salts or complexes) in combination with antibiotics. They have reported interesting advances on the antimycobacterial effects of organic compounds; and antibiotics^13–16^ in *M*. *tuberculosis* strain H37Rv, which is the most commonly used control for *M*. *tuberculosis* identification in the clinical and research laboratory setting, and drug-resistant clinical isolates of *M*. *tuberculosis*. In this study, we tested the antimycobacterial activity of transition-metals (CuSO_4_, AgNO_3_, ZnSO_4_, and NiSO_4_) in combination with antibiotics against first-line anti-tuberculosis drug resistant isolates.

## Results and Discussion

One of the central strategies of the tuberculosis control program is early detection of drug-resistant Mycobacterium tuberculosis strains. No clinical strains with a monoresistance profile to ethambutol (EMB) were found in the present study. It has been reported that the frequency EMB resistance is lower than that for other antimycobacterial agents. EMB is an alternative drug in the standard four-drug combination therapy since it prevents treatment failure in resistant strains to other antimicrobial agents (i.e. streptomycin) and avoids the risk of side effects^17^. Ziehl Neelsen stain showed that all the strains were acid-alcohol-resistant bacilli and they also presented slow growth, ability to produce niacin and nitrate reduction, which confirmed they were *M*. *tuberculosis* strains^18^.

We tested first-line TB drugs (INH, RIF, STR and EMB) and transition-metal salts (CuSO_4_, AgNO_3_, NiSO_4_ and ZnSO_4_) to determine MIC values in clinical strains of *M*. *tuberculosis*. The summary of the observed MIC values is displayed in Table 1.

**Table 1:** MIC values of first-line TB drugs and transition-metal salts in *M*. *tuberculosis* isolates.

		H37Rv	OxPs-22	OxPs-4	152589	OxPs-19
First-line TB drugs	STR	0.25 µg/ml	4.0 µg/ml	0.5 µg/ml	1.0 µg/ml	4.0 µg/ml
	INH	0.125 µg/ml	1.0 µg/ml	0.125 µg/ml	0.25 µg/ml	0.125 µg/ml
	RIF	0.062 µg/ml	2.0 µg/ml	2.0 µg/ml	0.125 µg/ml	0.125 µg/ml
	EMB	1.0 µg/ml	8.0 µg/ml	1.0 µg/ml	0.5 µg/ml	1.0 µg/ml
Transition-metal salts	CuSO_4_	35 µM	200 µM	200 µM	200 µM	250 µM
	AgNO_3_	20 µM	40 µM	25 µM	25 µM	25 µM
	NiSO_4_	200 µM	500 µM	500 µM	160 µM	140 µM
	ZnSO_4_	100 µM	>500 µM	>500 µM	400 µM	400 µM

### Antimycobacterial effect of transition-metal salts/first-line TB drugs combinatorial treatments

We tested the combination of transition-metal salts (that showed best inhibitory effect) with the corresponding drug to which each isolate was resistant, via a checkerboard assay, a widely used methodology to test synergistic effects between antimicrobial agents^19–22^.

Our results showed that the transition-metal salts/drugs combinations did not inhibit cell viability for strains OxPS-22 (Supplementary Fig. S1), OxPS-4 and OxPS-19 (Supplementary Fig. S2); nevertheless, the INH/AgNO_3_ combination showed a positive effect in strain 152589, since a reduction of 50% of the MIC of both treatments was achieved, from 0.25 µg/ml and 25 µM to 0.125 µg/ml and 12.5 µM, respectively. Regarding strain H37Rv, the INH/AgNO_3_ combination showed the same effect observed in strain 152589 (Supplementary Fig. S3).

To determine the interaction between two or more drugs intended to be used in combination, the Fractional Inhibitory Concentration (FIC) Index was used^23,24^. It was interpreted as follows: FIC index of 0.5 was considered for synergism, FIC index of 1 was defined as additive effect, and antagonism when FIC index was 2 or 4. For INH/AgNO_3_ combinatorial treatment the FIC was 1 which means an additive effect. Moreover, the effect of the INH/AgNO_3_ combination in strains 152589 and H37Rv was found to be bactericidal.

The INH/AgNO_3_ combination can be considered as a potential antimycobacterial agent since we have shown it can inhibit bacterial growth at a lower concentration than each one as a separate treatment. These would allow decreasing in dose both, isoniazid and AgNO_3_, and at the same time reduce the potential toxic effects in mammalian cells. Some other authors have reported the use of transitions metals/drug complexes as antibacterial agents since the combination improves its antimycobacterial activity^25–28^.

It is well known that silver affects the permeability of the bacterial membrane^29^, whereas isoniazid is a pro-drug that requires activation by the catalase/peroxidase enzyme KatG, encoded by the *KatG* gene to exert its effect^30^. The active form acts by inhibiting the synthesis of mycolic acid through the NADH-dependent enoyl-acyl carrier protein (ACP)-reductase^31^. Nevertheless, the mechanism through which the INH/AgNO_3_ combination affects the cell viability in an INH-resistant strain has not been elucidated.

Silver induces the folding of proteins that are secreted from the cytoplasm and transported to the outer membrane which can lead to membrane destabilization and increased permeability. Our research group has previously reported^7^ that the combination of sublethal concentrations of antibiotics with silver salts alters multiple cellular processes, including the formation of disulfide bonds, central metabolism, iron homeostasis, and these changes are associated with an increase in the production of ROS and permeability of the bacterial membrane. Therefore, we hypothesize that the addition of sublethal doses of silver to antibiotic induces a marked increase in ROS, where silver contributes to the production of ROS, oxidative stress and bacterial cell death.

**Figure 1:**
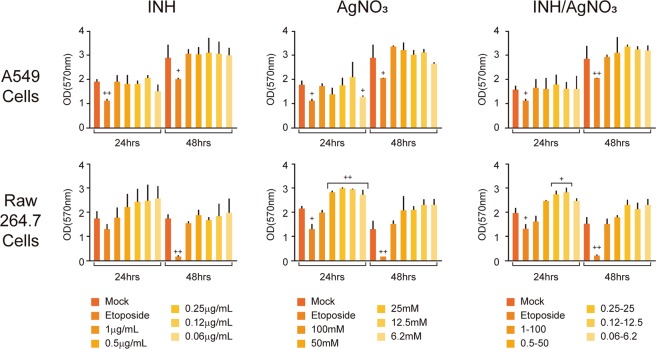
Effect of isoniazid, AgNO_3_ and the combinatorial treatment on cell viability of murine RAW 264.7 macrophages and human A549 lung cells. Cell viability was determined using the MTT assay at 24 and 48 h. Results are expressed as mean values ± SD (three independent experiments, three replicates per experiment at each concentration). INH (isoniazid), AgNO_3_ (silver nitrate), mock (untreated control), Etoposide (positive toxicity control). +y++ p < 0.05 was considered statistically significant. + refers to statistical significance with mock and ++ refers to statistical significance between the same time period.

As a complementary study, cytotoxic effects at 24 and 48 h treatment with isoniazid, silver nitrate and the combinatorial treatment were tested in two relevant respiratory cells lines. The results of the cytotoxic evaluation of INH, AgNO_3_ and the combinatorial treatment on cell viability of murine RAW 264.7 macrophages and human A549 lung cells is presented in Fig. 1. Our results indicate that INH did not induce any toxic effect on the cell viability of both A549 and RAW 264.7 cells after 24 and 48 h treatment. INH/AgNO_3_ combinatorial treatment did not increase significantly cell viability in A549 cells. Concerning RAW 264.6 cells, a significant increase of cell viability was observed upon exposure to 50–6.2 mM AgNO_3_ at 24 h (30% increase; p < 0.05). We observed same effect when RAW 264.7 cells were treated for 24 h with 0.25ug/mL-25mM and 0.12ug/mL-12.5 mM, INH/AgNO_3_ respectively. Nevertheless, we did not observe changes in growth after 48 h. This effect could be related to cellular stress which increased oxidative metabolism. Since the MTT assay is an assay for assessing cell metabolic activity, this would explain the observed increase in RAW 264.7 cells. These results suggest that INH, AgNO_3_ and combinatorial treatment have no toxic effect on A549 lung epithelial cells and RAW 264.7 macrophage cells.

## Conclusions

Mono-resistance to isoniazid is the most common first-line drug resistance in tuberculosis; therefore, it has been a challenge the development of more efficient and effective antimycobacterial drugs that show less toxicity against mammalian cells. Here, we have identified a combination of compounds (isoniazid/AgNO_3_) with antimycobacterial activity against an isoniazid-resistant clinical strain (strain 152589) of *M*. *tuberculosis*. The combinatorial treatment had a significant additive effect at 0.125 µg/ml isoniazid and 12.5 µM AgNO3 (50% decrease of individual MICs) and *in vitro* evaluation of cytotoxicity in RAW 264.7 and A549 cells showed no toxic effects. We have previously described transition-metals key role in several cellular processes and their antimicrobial effects; therefore, the combination of antibiotics with transition metals results in an excellent treatment alternative since antibacterial effect is enhanced with the combination of drugs, the concentrations used of both drugs are reduced, and the possibility of adverse effects is considerably reduced, resulting in a better outcome for the patient. Thus, this combination could be used in the development of new strategies to treat resistant strains of *Mycobacterium tuberculosis*.

## Methods

### Biosafety criteria and Microorganisms

All procedures involving *M*. *tuberculosis* specimens were carried out in a Class II A2 biological safety cabinet in a BSL-2 level containment facility located at the Regional Center for the Control of Infectious Diseases (CRCEI), Faculty of Medicine, Autonomous University of Nuevo León. Twelve strains with previous report of resistance to first-line drugs were selected from databases from the Regional Center for the Control of Infectious Diseases (CRCEI) from 2013 to 2016 (OxPs-22, 152589, 151655, OxPs-13, OxPs-3, 151228, OxPs-4, 160206, 141206, 160251, OxPs-19, 141406). *Mycobacterium tuberculosis* H37Rv strain was also included and it served as a drug-sensitive control. Ethical approval for this study was obtained from Ethics, Research, and Biosafety Committee from Faculty of Medicine, Autonomous University of Nuevo León.

### Strains reactivation and biochemical characterization

The selected strains were reactivated in Middlebrook 7H9 broth added with OADC (oleic acid, albumin, dextrose and catalase) at 10%, 5% Tween 80 and 0.2% glycerol. After their reactivation, Ziehl-Neelsen stain was performed to identify acid-alcohol-resistant bacilli. The twelve reactivated strains were phenotypically identified using biochemical assays of niacin accumulation and nitrate reduction previously described by Bernardelli *et al*.^18^. A positive control (*M*. *tuberculosis* H37Rv) and negative control (a tube with broth without inoculum) were included.

### Drug susceptibility testing

Antimicrobial susceptibility test for STR, INH, RIF, EMB was carried out by triplicate using critical concentration and minimum inhibitory concentration categories according to the recommendations of the World Health Organization in its technical report on the critical concentrations for drug susceptibility tests in medicines used in the treatment of drug-resistant tuberculosis^32^. The critical concentration of INH, RIF, EMB and STR were 0.2, 40, 4.0 and 2.0 μg/mL, respectively. The breakpoint concentrations (µg/ml) for resistance to STR, INH, RIF and EMB were defined as ≥4, 0.25, ≥0.5 and ≥4, respectively. Based on these results, from the twelve initially reactivated strains we selected strain 152589 (resistant to ISO), OxPs-4 (resistant to RIF), OxPs-19 (resistant to STR), and OxPs-22 (Multidrug-resistant; MDR) for subsequent experiments since they showed the resistance profile required. The rest of the strains were found to be sensitive therefore they were not considered for further analysis.

### Determination of MIC for antibiotics and transition-metal salts

MICs values were obtained using microplate Alamar Blue assay (MABA) as previously described^33^. Streptomycin, isoniazid, rifampicin and ethambutol, and metal salts: silver nitrate (AgNO_3_), copper sulfate (CuSO_4_), nickel sulfate (NiSO_4_) and zinc sulfate (ZnSO_4_) were evaluated. Clinic strains cultures were subcultured in Middlebrook 7H9 broth (added with 0.2% glycerol, 0.05% Tween 80 and 10% OADC) for 21 days at 37 °C. The resulting mycobacterial suspension was adjusted at a turbidity of 1.0 McFarland standard and then diluted 1:25 in Middlebrook 7H9 broth (added with 0.2% glycerol and 0.05% OADC). Antibiotics or transition-metal salts were aliquoted (100 μl) into the first row of wells of a 96-well microtiter plate in which wells were prefilled with 100 μl of Middlebrook 7H9 broth (added with 0.2% glycerol and 0.05% OADC), row 1 wells were mixed 8 to 10 times using a pipettor. Then, 100 μl was withdrawn and transferred to row 2. Row 2 wells were mixed 8 to 10 times, followed by a 100-μl transfer from row 2 to row 3. This procedure was used to serially dilute the rest of the rows of the microtiter plate. Finally, 100 μl of 1:25 diluted mycobacterial suspension was added into the wells containing specified antibiotic. The microtiter plate was incubated at 37 °C for 5 days. The range of antibiotic concentrations used for determining MICs were: STR 0.125–4.0 μg/ml, INH 0.031–1.0 μg/ml, RIF 0.062–2.0 μg/ml, EMB 0.5–16 μg/ml. The range of metal salts concentrations used for determining MICs were: CuSO_4_: 15.625–500 μM, AgNO_3_: 15.625–500 μM, ZnSO_4_: 15.625–500 μM and NiSO_4_: 15,625–500 μM. On day 5, 20 μl of Alamar Blue (AB) solution and 12 μl of 10% sterile Tween 80 were added to the drug controls ((Middlebrook 7H9 broth with antibiotic), to the negative control (Middlebrook 7H9 broth alone) and to the positive control (Middlebrook 7H9 broth with mycobacterial suspension). The microtiter plate was placed at 37 °C for 24 hours. After incubation, the drug controls and the negative control displayed no color change, while the positive control changed to pink, then we added 20 μl of AB solution and 12 μl of 10% sterile Tween 80 into the remaining wells and incubated again at 37 °C for 24 hours. The results were interpreted as non-viable cells if they displayed no color change or viable if they had the same intensity as the control well at 1%, or any shade of pink, violet or purple.

### Determination of MIC for transition-metals/antibiotics combinatorial treatments

Checkerboard assays^19–22^ were performed in 96-well polystyrene plates, in order to determine the antimycobacterial effects of transition-metals salts and first-line TB drugs. The MIC fractions (0, 0.5, 0.25, and 0.125) of transition-metals salts were prepared along the abscissa axis of the plate and the first-line TB drugs MIC fractions (0, 0.5, 0.25, and 0.125) were placed along the ordinate axis of the plate. Concentrated transition-metals salts (4×) and first-line TB drugs solutions (8×) were prepared in culture media, so that when added to the culture the needed volume of transition-metals salts, first-line TB drugs and inoculum MICs fractions was reached.

Clinical isolates cultures were grown for 21 days at 37 °C in Middlebrook 7H9 broth (added with 0.2% glycerol, 0.05% Tween 80 and 10% OADC) for 21 days at 37 °C. The resulting mycobacterial suspension was adjusted at a turbidity of 1.0 McFarland standard and then diluted 1:25 in Middlebrook 7H9 broth (added with 0.2% glycerol and 0.05% OADC). MIC fractions of 0.5, 0.25 and 0.125 of each transition-metal salt and first-line TB drug nominal concentrations were combined to achieve final concentrations in a final volume of 200 μL, including the bacteria inoculum. These combinations were diluted 2-fold and 100 μL were transferred each time after a thorough mixing and discarding the last 100 μL from the 3^rd^ dilution. The 96-well plates were incubated at 37 °C for 5 days. After incubation, 20 μl of AB solution and 12 μl of 10% sterile Tween 80 were added into drug control, transition-metal salt control, and negative and positive control. Plates were incubated again at 37 °C for 24 hours, after that, drug control, transition-metal salt control, and negative displayed no color change, while the positive control changed to pink, then we added 20 μl of AB solution and 12 μl of 10% sterile Tween 80 into the remaining wells and incubated again at 37 °C for 24 hours. The results were interpreted as non-viable cells if they displayed no color change or viable if they had the same intensity as the control well at 1%, or any shade of pink, violet or purple. The optical density (OD) of the control and the treated inoculums were measured, and the respective values were recorded. All tests and their respective control samples were performed in replicates of three.

### Cell culture

Murine RAW 264.7 macrophages (ATCC TIB-71) and human A549 lung cells (ATCC CCL-185) were maintained in Advanced DMEM/F12 medium supplemented with 1% GlutaMax, 1% antibiotic/antimycotic and 3.5% FBS, all from GIBCO. Cells were cultured at 37 °C in a humidified atmosphere containing 5% CO2.

### Assay for cell viability

The effect of compounds in cell viability was determinate using MTT assay according to Freshney^34^. Briefly, RAW 264.7 or A549 cells (1 × 103 cell/ well in 200 µL of medium) were seeded in a 96-well plate and incubated for 24 h, the cells were treated with serial dilution of isoniazide (from 1 to 0.06 mg/mL), AgNO3 (from 100 to 6 mM) prepared in PBS, a mix of both compounds, or etoposide (20 μM) and incubated for 24 or 48 h. Medium was changed and 0.2 ml of medium with 0.5 mg of MTT was added to each well, and the cells were incubated for another 4 h. The optical density was measured at 570 nm on a microplate reader.

### Statistical analysis

The results were obtained at least by three independent experiments and are presented as means ± SD. Statistical analyses were performed by one-way analysis of variance (ANOVA) followed by t Student´s test. All statistical analyses were performed using the GraphPad Prism®, version 6.0 software. P values ˂ 0.05 were considered to indicate statistical significance.

## Supplementary information

Supplementary Data

## References

[1] World Health Organization. Global tuberculosis report 2017. WHO/HTM/TB/2017.23.

[2] Sloan DJ, Davies GR, Khoo SH (2013). Recent advances in tuberculosis: New drugs and treatment regimens. Curr. Respir. Med. Rev..

[3] Morones JR (2005). The bactericidal effect of silver nanoparticles. Nanotechnology..

[4] Morones-Ramirez JR (2009). El uso de la Plata en los antibióticos del futuro. Revista Digital Universitaria..

[5] Morones-Ramirez JRP (2010). metal precioso con amplio espectro de aplicaciones. Revista Ciencia y Desarrollo..

[6] Morones-Ramirez JR (2010). Historia de la plata: Su impacto en las antiguas civilizaciones y la sociedad moderna. Revista Digital Universitaria..

[7] Morones-Ramirez JR, Winkler JA, Spina CS, Collins JJ (2013). Silver enhances antibiotic activity against gram-negative bacteria. Sci Transl. Med..

[8] Pal S, Yoon EJ, Park SH, Choi EC, Song JM (2010). Metallopharmaceuticals based on silver (I) and silver (II) polydiguanide complexes: activity against burn wound pathogens. J. Antimicrob. Chemother..

[9] Pal S, Tak YK, Han E, Rangasamy S, Song JM (2014). A multifunctional composite of an antibacterial higher-valent silver metallopharmaceutical and a potent wound healing polypeptide: a combined killing and healing approach to wound care. New J. Chem..

[10] Rigo C (2012). Characterization and evaluation of silver release from four different dressings used in burns care. Burns..

[11] Ray S (2007). Anticancer and antimicrobial metallopharmaceutical agents based on palladium, gold, and silver N-heterocyclic carbene complexes. JACS..

[12] Mjos KD, Orvig C (2014). Metallodrugs in medicinal inorganic chemistry. Chem. Rev..

[13] Pereira GA (2012). A broad study of two new promising antimycobacterial drugs: Ag(I) and Au(I) complexes with 2-(2-thienyl)benzothiazole. Polyhedron..

[14] Mandewale. M, Thorat B, Shelke D, Yamgar R (2015). Synthesis and biological evaluation of new hydrazone derivatives of quinoline and their Cu(II) and Zn(II) complexes against *Mycobacterium tuberculosis*. Bioinorg. Chem. Appl..

[15] Dalecki AG (2015). Disulfiram and copper ions kill Mycobacterium tuberculosis in a synergistic manner. Antimicrob. Agents Chemother..

[16] Jafari A, Mosavari N, Movahedzadeh F, Jafari NS (2017). Bactericidal impact of Ag, ZnO and mixed AgZnO colloidal nanoparticles on H37Rv *Mycobacterium tuberculosis* phagocytized by THP‐1 cell lines. Microb. Pathog..

[17] Ahmad S, Jaber AA, Mokaddas E (2007). Frequency of embB codon 306 mutations in ethambutol-susceptible and -resistant clinical Mycobacterium tuberculosis isolates in Kuwait. Tuberculosis..

[18] Bernardelli, A. *Manual de Procedimientos*. *Clasificación fenotípica de las micobacterias*. Dirección de Laboratorio y Control Técnico. Available on:http://www.senasa.gov.ar/Archivos/File/File1443- mlab.pdf-BioSource (2007).

[19] Bajaksouzian S, Visalli MA, Jacobs MR, Appelbaum PC (1997). Activities of levofloxacin, ofloxacin, and ciprofloxacin, alone and in combination with amikacin, against acinetobacters as determined by checkerboard and time-kill studies. Antimicrob. Agents Chemother..

[20] Sweeney MT, Zurenko GE (2003). *In vitro* activities of linezolid combined with other antimicrobial agents against staphylococci, enterococci, pneumococci, and selected gram-negative organisms. Antimicrob. Agents Chemother..

[21] Orhan G, Bayram A, Zer Y, Balci I (2005). Synergy tests by E test and checkerboard methods of antimicrobial combinations against Brucella melitensis. J. Clin. Microbiol..

[22] Santos DA, Nascimento MM, Vitali LH, Martinez R (2013). *In vitro* activity of antimicrobial combinations against multidrug-resistant Pseudomonas aeruginosa. Rev. Soc. Bras. Med. Trop..

[23] Berenbaum MC (1978). A method for testing the synergy with any number of agents. J. Infect. Dis..

[24] Eliopoulos GM (1989). Synergism and antagonism. Infect. Dis. Clin. North Am..

[25] Sen C (2018). Platinum (II)-azoimidazole drugs against TB and cancer: structural studies, cytotoxicity and anti-mycobacterial activity. Polyhedron..

[26] Pati R, Sahu R, Panda J, Sonawane A (2016). Encapsulation of zinc-rifampicin complex into transferrin-conjugated silver quantum-dots improves its antimycobacterial activity and stability and facilitates drug delivery into macrophages. Sci. Rep..

[27] Patil SD (2013). Synthesis and antimycobacterial activity of Cu (II) complexes containing thiosemicarbazones ligand. Der. Pharmacia Sinica..

[28] Poggi M (2013). New isoniazid complexes, promising agents against *Mycobacterium tuberculosis*. J Mex Chem Soc..

[29] Gurunathan S, Han JW, Kwon DN, Kim JH (2014). Enhanced antibacterial and anti-biofilm activities of silver nanoparticles against Gram-negative and Gram-positive bacteria. Nanoscale Res. Lett..

[30] Zhang Y, Heym B, Allen B, Young D, Cole S (1992). The catalase-peroxidase gene and isoniazid resistance of *Mycobacterium tuberculosis*. Nature..

[31] Rawat R, Whitty A, Tonge PJ (2003). The isoniazid-NAD adduct is a slow, tight-binding inhibitor of InhA, the *Mycobacterium tuberculosis* enoyl reductase: Adduct affinity and drug resistance. Proc. Natl. Acad. Sci. USA.

[32] World Health Organization. Technical report on critical concentrations for drug susceptibility testing of medicines used in the treatment of drug-resistant tuberculosis. WHO/CDS/TB/2018.5.

[33] Palomino JC (2002). Resazurin microtiter assay plate: simple and inexpensive methods for detection of drug resistance in *Mycobacterium tuberculosis*. Antimicrob. Agents Chemother..

[34] Freshney, R. I. *Culture of Animal Cells*. *A manual of basic technique* (ed. Wiley and Son) (New York 2005).

