# Supplementary material for: Antibacterial Activity of combinatorial treatments composed of transition-metal/antibiotics against Mycobacterium tuberculosis
Source: Sci Rep. 2019 Apr 2;9:5471. doi: 10.1038/s41598-019-42049-5 (PMC6445279; doi:10.1038/s41598-019-42049-5)
Supplement: Supplementary file 1 — Supplementary Data [file 41598_2019_42049_MOESM1_ESM.docx]

**Supplemental Data**

**Antibacterial Activity of combinatorial treatments composed of transition-metal/antibiotics against *Mycobacterium tuberculosis***

Montelongo-Peralta L. Z.^1,2^, León-Buitimea A.^1,2^, Palma-Nicolás J. P.^3^, Gonzalez-Christen J.^4^, Morones-Ramírez J. R.^1,2, *^

^1^Universidad Autónoma de Nuevo León, UANL. Facultad de Ciencias Químicas. Av. Universidad s/n. CD. Universitaria, 66455, San Nicolás de los Garza, NL, México; ^2^Centro de Investigación en Biotecnología y Nanotecnología, Facultad de Ciencias Químicas, Universidad Autónoma de Nuevo León. Parque de Investigación e Innovación Tecnológica, Km. 10 autopista al Aeropuerto Internacional Mariano Escobedo, 66629, Apodaca, Nuevo León, México; ^3^Centro Regional de Control de Enfermedades Infecciosas, Facultad de Medicina, Universidad Autónoma de Nuevo León, UANL. Av. Madero and Dr. Aguirre Pequeño s/n, Mitras centro, 64460, Monterrey, Nuevo León, México. ^4^Laboratorio de Inmunidad Innata. Facultad de Farmacia. Universidad Autónoma del Estado de Morelos. Av. Universidad 1001, Col. Chamilpa, 62209, Cuernavaca, Morelos, México.

* Corresponding author: José Rubén Morones-Ramírez.

Universidad Autónoma de Nuevo León, UANL. Facultad de Ciencias Químicas. Av. Universidad s/n. CD. Universitaria, 66455, San Nicolás de los Garza, NL, México

Tel: +52-818-329-4000 Ext 3439

Email: [jose.moronesrmr@uanl.edu.mx](mailto:jose.moronesrmr@uanl.edu.mx); [morones.ruben@gmail.com](mailto:morones.ruben@gmail.com)


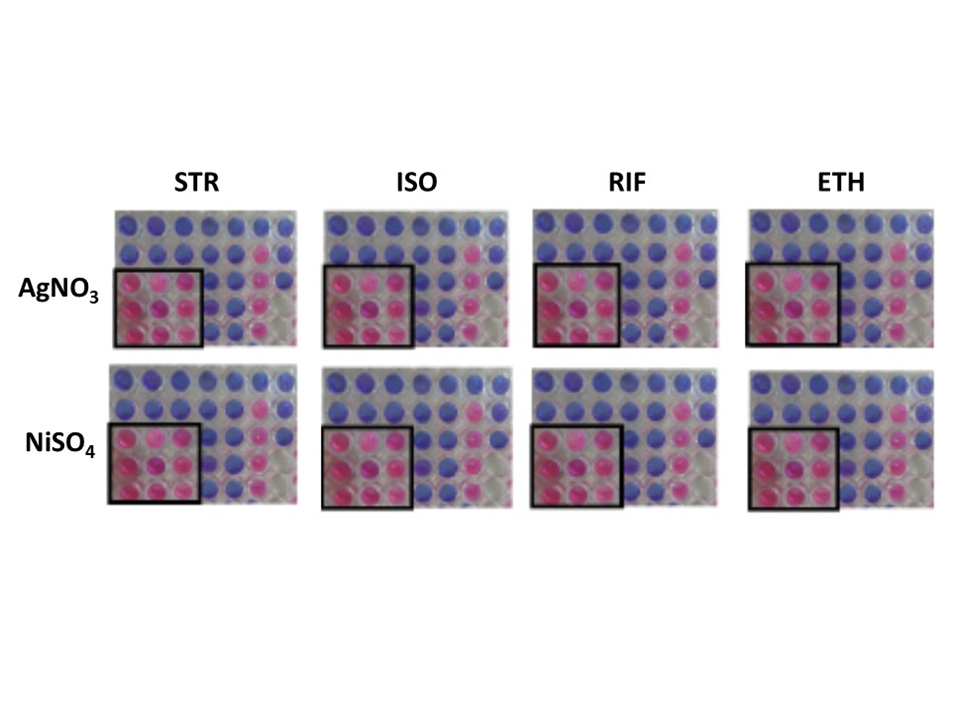


**Supplementary Figure S1.** Antimycobacterial activity of MIC fractions of transition-metal salts and drugs against strain OxPs-22 of *Mycobacterium tuberculosis* via checkerboard assay. Combinatorial treatments of STR with AgNO_3_ and NiSO_4_, INH with AgNO_3_ and NiSO_4_, RIF with AgNO_3_ and NiSO_4_, and EMB with AgNO_3_ and NiSO_4_ were tested. No growth inhibition was observed at any of the combinatorial treatments. Each checkerboard treatment was performed in triplicates. Black boxes show the combinatorial area of interest.


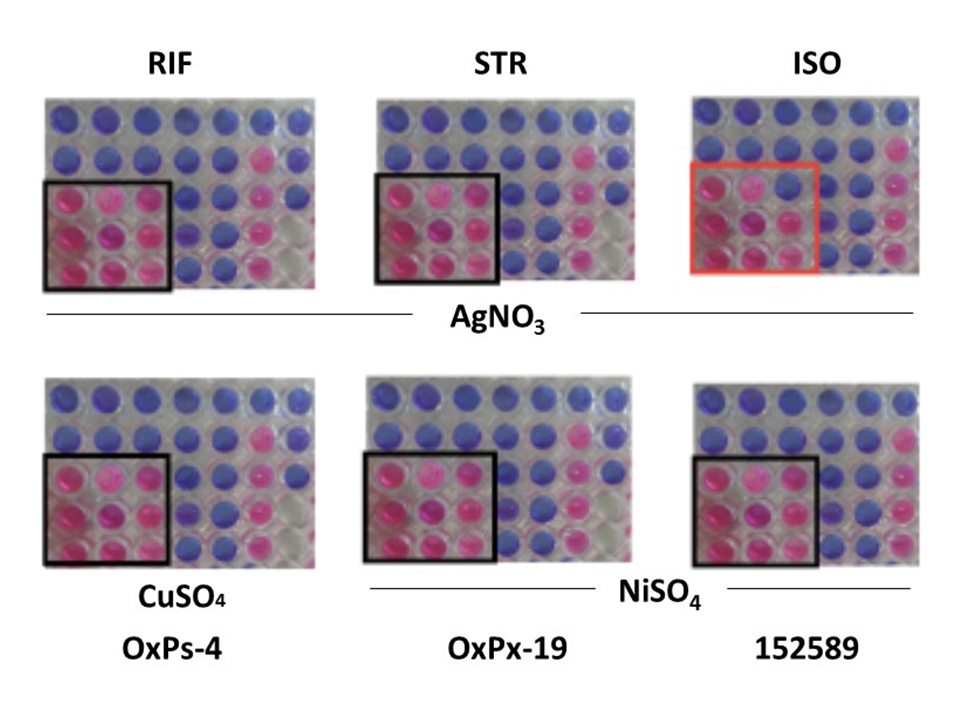


**Supplementary Figure S2.** Inhibitory effect of the MIC fractions of transition-metal salts and drugs against strains OxPs-4, OxPx-19 and 152589 of *Mycobacterium tuberculosis* via checkerboard assay. Experiments with combinatorial treatments of RIF with AgNO_3_ (A1) and CuSO_4_ (A2), STR with AgNO_3_ (B1) and NiSO_4_ (B2), INH with AgNO_3_ (C1) and NiSO_4_ (C2). Combinatorial treatments did not inhibit cell viability for strains OxPS-4 and OxPS-19; nonetheless, the ISO/AgNO_3_ combination showed positive interaction in strain 152589 (red box). Each checkerboard treatment was performed in triplicates. Black boxes show the combinatorial area of interest. Red box shows area where growth inhibition was observed.


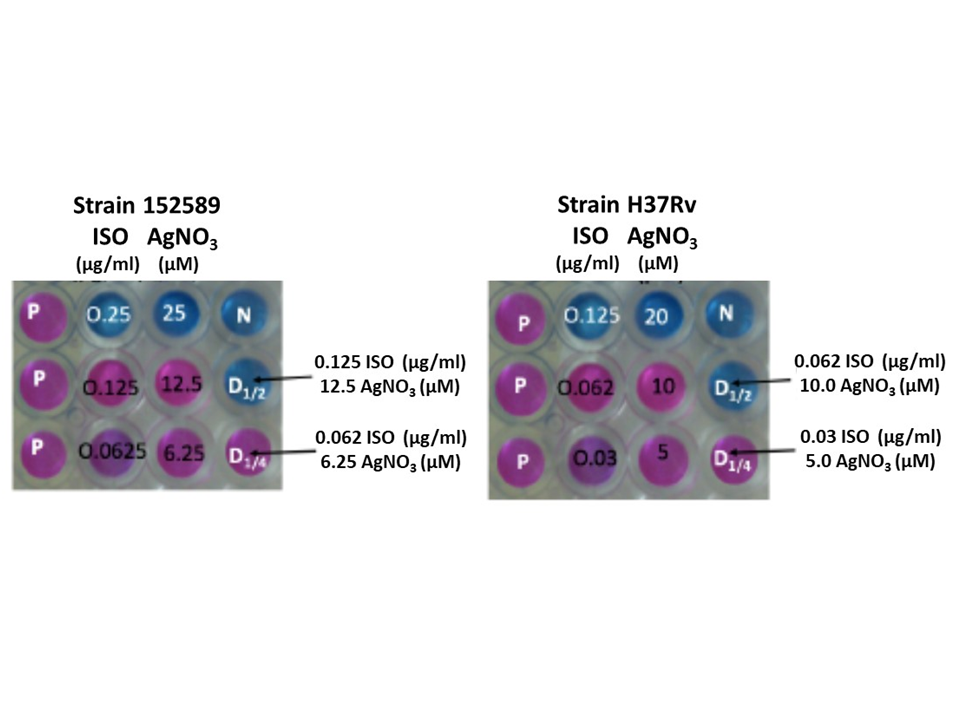


**Supplementary Figure S3.** Additive effect of INH/AgNO_3_ combinatorial treatment against strains 152589 and H37Rv of *Mycobacterium tuberculosis*. A reduction of 50% of the MIC of both treatments was achieved in strain 152589, from 0.25 µg/ml and 25 µM to 0.125 µg/ml and 12.5 µM, respectively. Same inhibitory effect was observed in strain H37Rv. Each experiment was performed in triplicates. INH= isoniazid, P= Positive growth control, N=Negative growth control, D_1/2_= ½ MIC, D_1/4_= ¼ MIC.
